# Low temperature cues ontogenetic shifts by multiple sizes of juvenile Atlantic tarpon (*Megalops atlanticus*) in restored mangrove creeks of south Florida

**DOI:** 10.1111/jfb.70145

**Published:** 2025-07-16

**Authors:** Matthew S. Kendall, Bethany L. Williams, Patrick M. O'Donnell

**Affiliations:** ^1^ NOAA/NOS/NCCOS Marine Spatial Ecology Division Silver Spring Maryland USA; ^2^ Rookery Bay National Estuarine Research Reserve Naples Florida USA

**Keywords:** construction, home area, nursery habitat, telemetry, temperature, water level

## Abstract

Juvenile tarpon [25–35 cm (TL)] implanted with transmitters moved into restored mangrove habitat within 3 days after construction. Emigration from juvenile habitat was significantly related to low temperatures <20°C but not water levels. There was overlap among cohorts with 50% of tarpon emigrating one winter and the remainder waiting a year later. Mangrove restoration should be scheduled to avoid blocking egress routes (e.g., silt curtains) when tarpon are likely to emigrate.

Despite their importance to recreational fisheries, many aspects of early life history of Atlantic tarpon (*Megalops atlanticus*, Valenciennes 1847) remain unresolved (Adams et al., 2014). For example, findings on the seasonal timing and cues for juvenile tarpon to leave their nursery habitat vary among studies. In the southeastern USA, tarpon use small mangrove‐lined tidal creeks, marshes or coastal ponds as juveniles, then undertake multiple ontogenetic shifts in nursery habitat, using progressively more open parts of estuaries as they grow larger (Elmo et al., 2021; Navarro‐Martínez et al., 2020; Rickards, 1968; Zerbi et al., 2005). Some evidence suggests that shifts are cued by low winter temperatures in the northern part of their range, as observed in Georgia (Rickards, 1968), South Carolina (Elmo et al., 2021; Mace et al., 2018) and Louisiana (Stein et al., 2016). Studies in other areas indicate that habitat shifts occur due to high‐water events such as storm surges in summer or fall, as observed in southwest Florida (Bunting et al., 2024; Wilson et al., 2019).

In addition to questions around the cues and timing of ontogenetic shifts, it should be recognized that nearly all tarpon nursery habitat lies within the footprint of coastal development. Small creeks, ponds and marshes are often reshaped, used as runoff catchments, managed for mosquito control or water fowl enhancement or otherwise disconnected from the open estuary by berms, roads and water control structures (Harrington & Harrington, 1982, Tyler et al. 1998, Mace et al., 2018, Elmo et al., 2021). Habitat connectivity in such areas can be restricted to only those times with higher tidal levels in certain seasons, during storm surge or as human‐operated water‐control structures, such as culverts, are opened and closed (Cianciotto et al., 2019; Poulakis et al., 2002; Wilson et al., 2019). With the recognition that these modified habitats can be essential to tarpon and other species (Adams et al., 2014), there is a growing emphasis on restoring their natural hydrology (Brockmeyer et al., 2022; Lewis & Gilmore, 2007). Indeed, further research is needed to determine the extent to which tarpon use restored habitats, whether steps can be taken to reduce impacts to tarpon during restoration, how quickly restored habitats are utilized once created, and whether restored areas ultimately contribute to adult populations. This study investigated these issues by evaluating the following: (1) use of a newly restored mangrove creek by juvenile tarpon, (2) timing and potential cues for ontogenetic habitat shifts (i.e., cold temperature and/or high‐water events), (3) size of the habitat area used and (4) the connectivity of the restored mangrove creeks through road culverts.

Nineteen juvenile tarpon (24–33 cm TL) were captured between 15 and 18 December 2022 and surgically implanted with coded acoustic transmitters (InnovaSea Systems Inc. model V8‐4L, 130–230 s ping rate, 448‐day battery) (Table 1). Fish movements were monitored using 14 acoustic data receivers (InnovaSea Systems Inc. model VR2W) deployed in a hydrologically restored mangrove creek that is part of Fruit Farm Creek (FFC) in southwest Florida (Figure 1a). Restoration consisted of installing new pipe culverts under a road and excavating creeks to enable tidal flow (Coastal Resources Group et al., 2023). Culverts were installed in November and December 2022 and were in place prior to tagging. Of note, the western culvert was installed and opened on 12 December 2022, creating small pools that previously did not exist adjacent to the road. Tarpon were caught and tagged in the pools 3 days after their construction was completed. Excavation of the eastern creek was completed before the tracking period. Excavation of the western creek occurred on 21 discontinuous days from 19 December 2022 to 23 January 2023 during the tracking period. Water was always present in culverts even during low tides, enabling tarpon to come and go at any time. All fish were captured and released on the upstream (i.e., south) side of the culverts, farthest from the open waters of the estuary. Three suspected post‐tagging mortalities provided little data and were not analysed. Water temperature (hereafter referred to as temperature) and height are from National Estuarine Research Reserve National Monitoring Program's Rookery Bay NERR Middle Blackwater River station (RKBMBWQ) (NOAA National Estuarine Research Reserve System 2025). Temperature was always within the tolerance reported for juvenile tarpon (Mace et al., 2017; Stein et al., 2016).

**TABLE 1:** Summary statistics for tarpon tracked at Fruit Farm Creek (FFC).

Fish ID	TL (cm) at tagging	Tagging date	Tagging culvert	Culvert transit dates	Movement speeds (m/s)	Departure date	Minimum temp. at departure (C)	Maximum water ht. at departure (m)	Estimated size TL at departure (cm)	Home creek length (linear m)
MEAT 625	26	15‐Dec‐2022	East	22‐Dec‐2022	N/A	22‐Dec‐22	21.0	1.8	27	N/A
MEAT 632	27	15‐Dec‐2022	East	22‐Dec‐2022	N/A	22‐Dec‐22	21.0	1.8	28	N/A
MEAT 624	26	15‐Dec‐2022	East	25‐Dec‐2022	N/A	25‐Dec‐22	13.3	1.7	27	N/A
MEAT 640	24	17‐Dec‐2022	West	20‐Dec‐2022 21‐Dec‐2022 24‐Dec‐2022 24‐Dec‐2022	0.04	25‐Dec‐22	13.3	1.7	25	N/A
MEAT 645	26	17‐Dec‐2022	East	25‐Dec‐2025	0.03	26‐Dec‐22	12.1	1.7	27	N/A
MEAT 651	27	17‐Dec‐2022	East	23‐Dec‐2022 24‐Dec‐2022 26‐Dec‐2022	0.02, 0.02, 0.74	26‐Dec‐22	12.1	1.7	28	N/A
MEAT 626	24	17‐Dec‐2022	West	18‐Dec‐2022 24‐Dec‐2022 24‐Dec‐2022 5‐Jan‐2022	0.03	11‐Jan‐23	19.6	1.5	26	N/A
MEAT 598	27	17‐Dec‐2022	West	19‐Dec‐2022	0.13, 0.32	19‐Jan‐23	17.8	1.8	29	N/A
MEAT 634	27	18‐Dec‐2022	West	22‐Dec‐2025	0.05	12‐Dec‐23	18.8	1.5	54	210
MEAT 596	33	17‐Dec‐2022	West	19‐Dec‐2022 20‐Dec‐2022 20‐Dec‐2022 21‐Jan‐2023 15‐Dec‐2023	0.13, 0.34	15‐Dec‐23	19.6	1.5	60	320
MEAT 649	29	18‐Dec‐2022	West	18‐Dec‐2022 19‐Dec‐2022 25‐Dec‐2022 4‐Jan‐2024	0.02	04‐Jan‐24	18.7	1.4	58	320
MEAT 597	27	17‐Dec‐2022	West	21‐Dec‐2022 21‐Dec‐2022 25‐Dec‐2022 25‐Dec‐2022 24‐Jan‐2023	0.02	11‐Jan‐24	19.5	1.7	56	90
MEAT 604	27	17‐Dec‐2022	East	21‐Jan‐2024	N/A	21‐Jan‐24	17.6	1.3	57	25
MEAT 618	26	15‐Dec‐2022	East	25‐Dec‐2022 21‐Jan‐2022	0.07	21‐Jan‐24	17.6	1.3	56	25
MEAT 652	27	17‐Dec‐2022	East	5‐Feb‐2023 5‐Feb‐2023 29‐Jul‐2023 29‐Jul‐2023 21‐Jan‐2024	N/A	21‐Jan‐24	17.6	1.3	57	25
MEAT 603	27	17‐Dec‐2022	East	25‐Dec‐2022	0.02, 0.15	22‐Jan‐24	17.2	1.5	57	260
Mean	26.9		Mean	2.6	0.13				Mean	159
SE	0.5		Range	1–5	0.02–0.74				SE	47

*Note*: Movement speeds were calculated based on detections outside of home creek segments. Home creek segments (lengths) are the linear distance between receivers utilized excluding emigration periods. Departure date is the date of last detection. Potential size at emigration was calculated assuming a growth rate of 0.75 mm/day.

Abbreviations: N/A, available data insufficient to calculate a value; SE, standard error.

**FIGURE 1:**
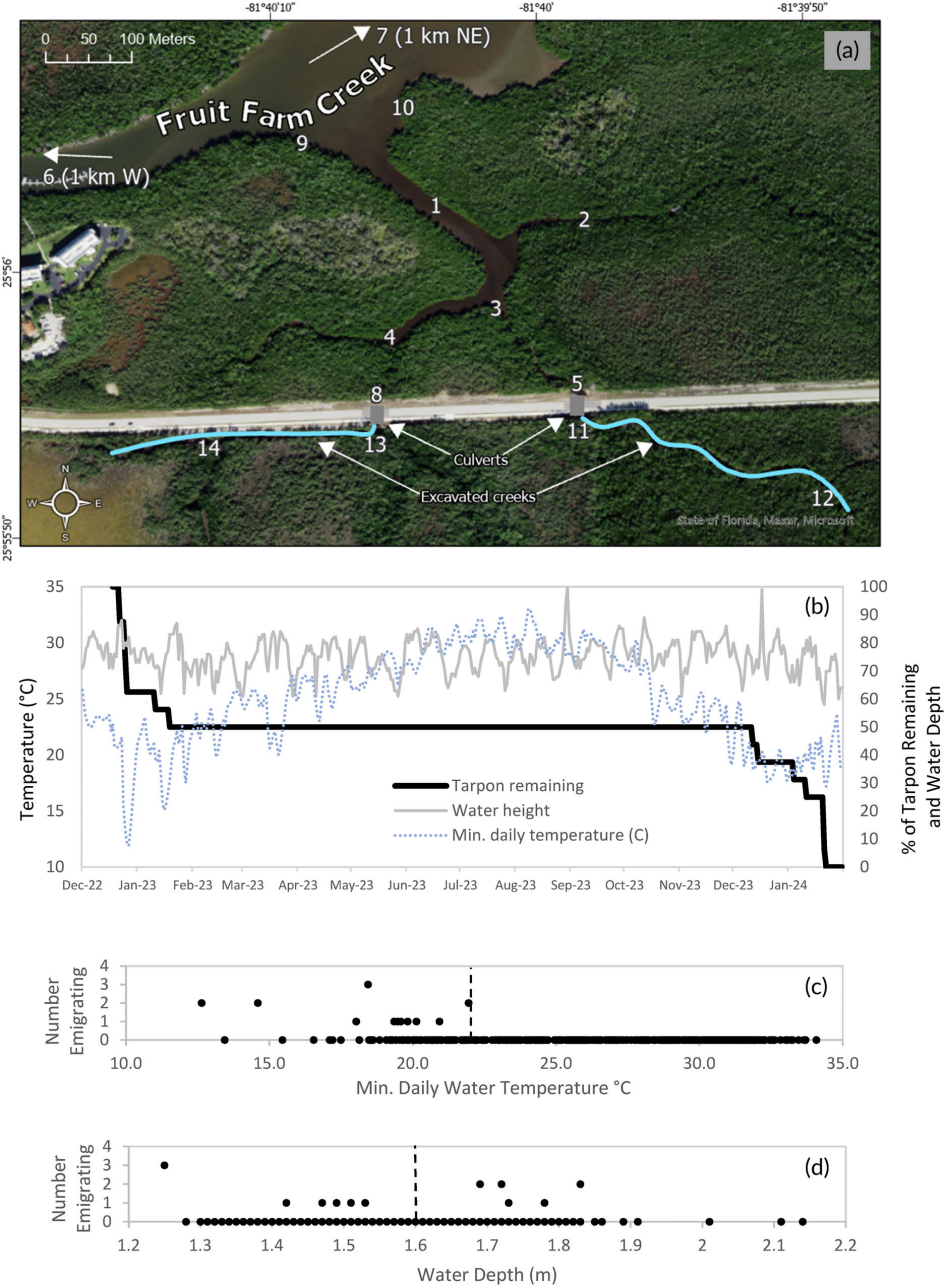
(a) Fruit Farm Creek (FFC) restoration study area east of Marco Island, Florida. Numbers denote position of receivers. (b) Percentage of tarpon remaining at FFC compared to water temperature and water height. Temperature shown is the daily minimum (°C). Water height shown is maximum daily water height expressed as the percentage of the highest value observed during the tracking period (i.e., 30 August 2023). (c) Daily water temperatures (°C) observed during the tracking period sorted from smallest to largest and the corresponding number of tarpon emigrating on those days. Dotted line is the 10th percentile of water temperature days. (d) Daily water depth (m) observed during the tracking period sorted from shallowest to deepest and the corresponding number of tarpon emigrating on those days. Dotted line is the 90th percentile of water height days.

Immediately after release, all tracked fish (*n* = 16) stayed at their tagging location for up to a week. This was followed by a period of widespread movements, beginning when temperatures dropped from 23 to 13°C between 22 and 26 December 2022 (Figure 1b). Movements included daily use of the main FFC tributary north of the culverts (i.e., receivers 1, 9 and 10; Figure 1a). Six fish never returned following this cold event, four of which were detected passing by outer receivers (i.e., receivers 6 or 7) as they departed. An additional two fish departed 3 weeks later, coincident with another cold snap on 16 January 2023 when water temperature reached 16°C. This initial period of widespread movements ended for all eight fish that remained in the study area at the same time as the second cold weather event concluded, specifically when temperatures rose above 20°C on 20 January 2023 (Figure 1b). These fish then remained in the study area near the culverts all year until temperatures dropped again the following December. All eight fish then ceased detection beginning in December 2023, with the largest number leaving in January 2024 when water temperatures dropped to 18°C (Figure 1b).

A permutation test was used to determine if this departure pattern on cold weather dates could have occurred by random chance (Good, 2005). For this test, the probability of tarpon randomly leaving when temperatures fell below some critical value was calculated. First, a table containing all 428 days of the tracking span was sorted from coldest to warmest, retaining the number of tarpon that emigrated on each day as a value in a second column (Figure 1c). The temperature of 22.0°C was selected as the critical value. This corresponded to the coldest 10% of tracking days and was the temperature below which all 16 tarpon emigrated. Next, the order of the second column (i.e., the number of tarpon emigrating on each day) was randomized, and the number of tarpon that randomly could have left on days when temperatures were < 22.0°C was recorded. This column was randomly sorted 1000 times, and the result was saved. The proportion of times that all 16 tarpon could have emigrated randomly when temperatures were <22.0°C was expressed as a *p*‐value. In this analysis, there was no detectable chance (*p* < 0.001) for this temperature‐related departure pattern to have arisen randomly.

Water depth, the other variable sometimes associated with tarpon movement, varies in the study area based on tide, wind speed and direction and atmospheric pressure. Range in maximum daily water depth was 0.89 m. Two dates had extremely high values compared to the rest of the tracking period: 30 August 2023 when Hurricane Idalia passed nearby, and 17 December 2023 during a period of strong onshore winds (Figure 1b). Other relatively high values occurred bi‐weekly during spring tides. Tracking data revealed that tarpon emigrated during nearly all water‐level conditions (Figure 1d). Most emigrated during moderate water level conditions, although three departed on 21 January 2024, the date with the very lowest water levels observed during the study. None departed during the highest water levels whether those occurred during summer or winter.

Permutation was also used to test the hypothesis that high‐water events were significantly related to tarpon emigration. Similar to the test for temperature, tracking days were sorted by maximum daily water level from lowest to highest, retaining the number of tarpon that emigrated on each day as a value in a second column (Figure 1d). The water level of 1.77 m was selected as the critical value, which corresponded to the top 10% of tracking days with highest water levels. Three of the tarpon emigrated at water levels above this value. The order of the second column was randomized 1000 times, and the number of tarpon that could have randomly left when water level above 1.77 m was recorded for each iteration. In this analysis, there was a 24% chance (*p* = 0.240) that three or more tarpon could have emigrated randomly when water level was above 1.77 m. In other words, there was no significant relationship between high‐water levels and emigration dates.

This suggests that low temperatures in winter months cue ontogenetic emigration for tarpon in this restored system rather than water level. Interestingly, cold‐related emigration is reported in the northern part of the tarpon's range (e.g., Georgia, South Carolina, Louisiana), wherein juveniles often leave nursery habitats in fall or winter as lethal temperatures regularly occur (Elmo et al., 2021; Mace et al., 2018; Rickards, 1968; Stein et al., 2016), but has not been reported in the warmer southern part of their range until this study. In most Florida studies, for example, this size class of tarpon has been reported departing nursery habitats primarily during summer or early fall coinciding with high‐water events (Bunting et al., 2024; Wilson et al., 2019). However, fish in most studied systems in Florida can only depart opportunistically during high water, as they are seasonally landlocked in coastal ponds (Bunting et al., 2024) or when a culvert is opened (Cianciotto et al., 2019). Results here suggest that the timing and cues for emigration in Florida depend on the specific landscape setting (e.g., fully open to tidal flow year‐round vs. seasonal opportunistic) and are not driven by only one type of cue or opportunity. Indeed, none of the juvenile tarpon in our study left in the summer or during particular water‐level conditions despite the culverts always being open to daily tidal flow.

Another possible influence on emigration considered here was excavation of the western creek, which occurred intermittently over the first month of the tracking period. Visual assessment of tarpon detections in abacus plots revealed that no individuals left the area of the western creek at times corresponding to excavation although detailed daily construction records were not kept, which prevented statistical analysis.

A last possibility for cessation of detection, at least for the eight fish that stayed in the restoration area for a whole year, is that the transmitter batteries died but the fish remained in the system. These were older units, and the manufacturer could not guarantee that their transmissions would not have ceased prior to their programmed end date in April 2024 (Innovasea Inc., personal communication). However, that two sets of fish departed synchronously when mean daily temperatures dropped below 20°C in December/January of two different years, and none left during summer, suggests that these were all emigration events cued by cold temperature rather than premature expiration of batteries.

Results here indicate that tarpon from the same year class departed these nursery creeks a year apart at two different sizes despite all having the opportunity to emigrate at any time. Estimated growth rates for tarpon range from 0.07 to 1.44 mm/day (Wilson et al., 2019) and depend on temperature, geographic region and habitat quality. Here, we used the midpoint of this range (i.e., 0.75 mm/day) to estimate potential length at emigration. The fish departing the same winter as tagging were of course a similar size as when they were tagged a few weeks earlier; however, those leaving a year later could have grown from 24 to 33 cm TL at the time of tagging to 54–60 cm by the time of emigration (Table 1), using this theoretical growth rate. Indeed, tarpon in the 40–50 cm size class were observed in June 2023 at the study site (M. Kendall personal observation), and multiple size classes have also been observed in the same habitat elsewhere (Bunting et al., 2024; Mace et al., 2018). It is also noteworthy that a large proportion of the juvenile tarpon tagged in some other studies were not detected leaving nursery habitats despite others in their cohort doing so (Bunting et al., 2024; Cianciotto et al., 2019; Wilson et al., 2019). Collectively, this indicates a more protracted and variable range of sizes and ages that juvenile tarpon use mangrove nurseries compared to species that use juvenile habitat for only a season or single year.

It was also of interest to determine if the 25‐m‐long culverts were an impediment to fish passage even though they always contained at least ~50 cm of water depth. All 16 tarpon conclusively moved through the culverts (i.e., detections occurred on one side of the road than the other) at least once [mean 2.6 crossings per fish +/− 0.4 standard error (SE)]. Of the 41 culvert crossings detected, all but 2 occurred during winter months. Those two crossings occurred in summer, with both taking place on 29 July 2023, by the same fish (MEAT 652, Table 1), a date with no temperature, depth or construction anomalies. Elsewhere in southwest Florida, juvenile tarpon were observed to emigrate through even narrower and longer culverts (i.e., 30 cm diameter and > 100 m long; Wilson et al., 2019). Overall, this indicates that culverts with similar dimensions do not pose a movement barrier to juvenile tarpon unlike some other fishes such as grey snapper (*Lutjanus griseus*, Linnaeus 1758) and hardhead catfish (*Ariopsis felis*, Linnaeus 1766) that utilize similar habitats (M. Kendall personal observation).

The network of receivers in the study also enabled the estimation of home creek extent for those eight tarpon that did not emigrate the first winter. Visual inspection of the abacus plots and measurements of the linear distance between receivers revealed that tarpon occupied a small segment of creeks, encompassing only 2–3 adjacent receivers, or up to 320 linear metres of mangrove‐lined creeks (mean 159 m ± 47 SE). There was no increase in space use over the year even though fish were older and probably increased in length.

There are several important implications from this study for planning or promoting mangrove restoration projects. First, juvenile tarpon can quickly move into restored mangrove habitats, even within a week of their construction, and not just by seasonal recruitment of postlarvae but also as juveniles in the 25–35 cm TL range. Second, they are not necessarily displaced by disturbance, including excavation of creek beds in their home creeks. Third, there is complete overlap among year classes present, which means there is no time when juveniles can be completely avoided (i.e., 1 year class completely emigrates the creeks before the next year class recruits). Fourth, juveniles that stayed for another year did not use progressively larger creek segments as they aged. Collectively, results demonstrate the immediate benefit of hydrologic restoration of mangrove creeks as habitat for juvenile tarpon and their potential contribution to adult populations via emigration. Care should be taken when scheduling restoration to not block egress routes during construction activities, such as by silt curtains or temporary construction dams, during cold months when tarpon are more likely to emigrate. It should also be noted that construction cannot be scheduled to completely avoid times when tarpon are present, as successive year classes overlap across seasons and years.

## AUTHOR CONTRIBUTIONS

Matthew S. Kendall, Bethany L. Williams, Patrick M. O'Donnell conducted field research, collected data, prepared and edited the manuscript.

## FUNDING INFORMATION

This study was funded by NOAA/NOS/NCCOS, Rookery Bay National Estuarine Research Reserve.
